# Research on the Defect Detection Method of Steel-Reinforced Concrete Based on Piezoelectric Technology and Weight Analysis

**DOI:** 10.3390/s25133844

**Published:** 2025-06-20

**Authors:** Yilong Yu, Yulin Dong, Yulong Jiang, Fan Wang, Qianfan Zhou, Panfeng Ba

**Affiliations:** 1 China State Construction Hongda Engineering Co., Ltd., Beijing 100016, China; ying.un@163.com (Y.Y.); wangfan@cohl.com (F.W.); zhouqianfan@cohl.com (Q.Z.); 2 School of Civil Engineering, Tianjin Chengjian University, Tianjin 300384, China; dongyl1851@163.com (Y.D.); hnpybpf-369@163.com (P.B.)

**Keywords:** steel-reinforced concrete, piezoelectric wave method, wavelet packet energy, analytic hierarchy process, damage metric

## Abstract

Aiming at the complex internal working conditions of steel-reinforced concrete structures, this paper proposes an active detection method for the internal hollow defects of steel-reinforced concrete based on wave analysis by using the driving and sensing functions of piezoelectric ceramic materials. The feasibility was verified through the single-condition detection test, revealing the propagation and attenuation characteristics of the stress wave signal under various detection conditions, and it was applied to the damage identification of steel-reinforced concrete rectangular section columns. Combined with the wavelet packet energy theory, the data processing of the original detection signal is carried out based on composite weighting by energy distribution entropy. Finally, the analytic hierarchy process (AHP) was introduced to study the weight vectors of different damage metrics on the detection signal, and a linear regression model based on different damage metrics was proposed as the comprehensive defect evaluation index. The research results show that the detection of internal defects in steel-reinforced concrete structures based on piezoelectric technology is applicable to concrete of different strength grades. With the increase of the detection distance and the degree of damage, the energy of the stress wave signal decreases. For example, under defect-free conditions, the energy value of the stress wave signal with a detection distance of 400 mm decreases by 92.94% compared to that with a detection distance of 100 mm. Meanwhile, it can be known from the defect detection test results of steel-reinforced concrete columns that the wavelet packet energy value under the defect condition with three obstacles decreased by 85.42% compared with the barrier-free condition, and the defect evaluation index (DI) gradually increased from 0 to 0.859. The comprehensive application of piezoelectric technology and weight analysis methods has achieved qualitative and quantitative analysis of defects, providing reference value for the maintenance and repair of steel-reinforced concrete structures.

## 1. Introduction

Due to its high rigidity and strong anti-deformation ability, steel-reinforced concrete can be used in the load-bearing parts of super high-rise buildings. However, due to the design of the internal rigid steel frame, the formation of cavities between the flange and the web, and the hardening and shrinkage of the concrete material, especially in areas where the concrete cannot enter under the transverse steel ribs, there may be peeling defects between the concrete and the steel ribs and web. This further reduces its mechanical properties and poses potential safety hazards [1,2]. In the past 10 years, many destructive and non-destructive testing methods have been developed in the engineering field to evaluate the health status of concrete structures, such as the core sampling method, impact echo method, and ultrasonic testing method [3,4]. However, the core drilling sampling method is a kind of damage detection, which cannot meet the requirements of the construction stage. The impact echo instrument used in the impact echo method is greatly affected by the size and shape of the structural members, and the effect is not good for detecting defects in the deeper parts. It is not suitable for the on-site compactness detection of the multi-chamber condition of the steel-reinforced concrete giant column. Ultrasonic testing can detect cracks of different sizes in steel-concrete composite structures. Its acoustic emission and digital image presentation techniques are used to identify concrete fracture parameters [5,6]. However, ultrasonic testing can only detect the internal defects of core concrete, and cannot effectively determine the steel-concrete separation defects due to poor cementation between the concrete and steel ribs. Traditional non-destructive testing methods still face limitations in providing real-time monitoring. Equipment sensors cannot approach key parts of the structure, and damage identification still requires qualified technicians to operate and study more concise and accurate damage characterization forms [6].

Piezoelectric ceramics are a type of information functional ceramic material capable of converting mechanical energy and electrical energy into each other. They have the advantages of fast response, high sensitivity, and the dual functions of sensing and driving [7,8,9]. Compared with the traditional method, the health detection method based on piezoelectric ceramics has the advantages of being real-time, sensitive, and not limited by the geometric shape of the test piece. Many scholars have made piezoelectric sensors used in the active health monitoring of civil engineering materials, such as concrete defects, internal damage of steel-reinforced concrete, grouting sleeve compactness, wood structure, carbon fiber-reinforced polymer (CFRP)-wood column structure, GFRP tube concrete column damage, and so on [10,11,12,13,14,15]. Among them, the quality defect detection method for concrete-filled steel tube is mainly studied by scholars at home and abroad. Wang et al. [16] developed a non-destructive detection method based on piezoelectric ceramic sensors and wave analysis to identify debonding defects in concrete-filled steel tube (CFST) structures. Maria C. Naoum [17] developed and validated a structural health monitoring system based on piezoelectric materials and electromechanical impedance technology, which can be used to detect the damage evolution of fiber-reinforced concrete under cyclic loading in real time, and provide data support for safety assessment and the reinforcement decision-making of existing buildings. Tan Kai Noel Quah [18] demonstrated the influence of defects on the stress wave field generated by the externally bonded piezoelectric ceramic sheet through experimental research and numerical simulation analysis. Maurya Krishna Kumar [19] embedded the piezoelectric sensor horizontally into the 3D-printed concrete layer, and studied the influence of different coating materials and thicknesses on the performance of the sensor. The piezoelectric sensing technology was used to realize real-time and non-destructive monitoring. The root mean square deviation was used to quantify the signal change, and the effectiveness of the detection technology was verified to solve the problem of high sensor embedding cost and short life cycles in traditional construction methods. Zhang et al. [20] realized the damage identification of L-shaped concrete-filled steel tubular columns under cyclic loading based on embedded piezoelectric ceramics. Zhang et al. [21] embedded four piezoelectric aggregate aggregates into the reinforced concrete beam sample, monitored its various regions through active monitoring technology, introduced the damage index to estimate the crack damage degree in the concrete structure, and verified the effectiveness of the proposed method through high-speed camera and image processing technology. Huang [22] evaluated the damage conditions of the edge nodes of the reinforced concrete frame through changes in signal amplitude and energy. It was found that the signal amplitude and energy were sensitive to the damage. According to the reasonable arrangement of the positions of the piezoelectric functional elements, the differences in the degree of damage at different parts of the component were identified. Wu et al. [23] developed tubular piezoelectric ceramic sensors for rapid sensor installation and two-dimensional monitoring of specimen damage, and applied them to the damage monitoring of asymmetric cross-shaped steel-reinforced concrete rectangular cross-section columns. Demi Ai et al. [24] proposed a new damage location method based on the combination of probability-weighted imaging algorithm and electromechanical admittance (EMA) technology, which is used to accurately locate the crack damage of reinforced concrete slabs and realize the millimeter-level location of cracks in reinforced concrete slabs. The above research shows that intelligent piezoelectric materials can be widely used in material performance evaluation and the damage identification of concrete structures. However, the process of structural health detection includes three aspects: structural detection, signal processing, and damage identification. At present, there have been many forward-looking achievements in the use of piezoelectric sensors to realize structural health detection. However, in the aspect of signal processing, the resonance peak of the response spectrum of the original detection signal and the wavelet packet energy of the signal are often selected as the characteristics of structural state change. Currently, there have been many forward-looking achievements in the realization of structural health detection using piezoelectric sensors. However, in terms of signal processing, the resonant peak of the response spectrum of the original detection signal and the energy of the signal wavelet packet are often selected as the characteristics of structural state changes [25]. In scenarios where the noise energy at the construction site is high but disordered, it is easy to cause misjudgment of the structural state. In terms of damage identification algorithms, most scholars adopt a single damage factor to measure the degree of time-domain signal variation of stress waves as a measure of the degree of structural damage [26].

**Figure 1:**
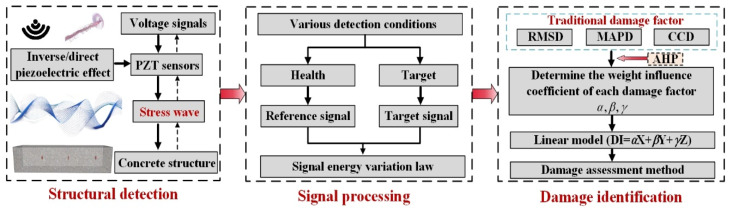
Technical route.

The research route of the steel-reinforced concrete defect detection method based on piezoelectric technology and weight analysis is shown in Figure 1. The purpose of this paper is to explore the different response effects of piezoelectric ceramics on various influencing factors inside steel-reinforced concrete structures through the analysis and application of the piezoelectric wave method and to reveal the propagation law of stress waves under different working conditions. A comprehensive correction coefficient method based on the sub-band energy ratio and entropy normalization is proposed to process and analyze the wavelet packet energy value of the original detection time-domain signal. The energy value is used to characterize the strength of the vibration spectrum signal and reflect the degree of signal attenuation under different working conditions. The damage metrics in active structural health detection are studied. Based on the existing typical damage metrics, the test and calibration are carried out. Combined with the influence of different damage metrics on the overall change in signal amplitude and vibration spectrum, a comprehensive damage evaluation index based on weight vectors of different damage metrics is proposed. The piezoelectric sensor is applied to the steel-reinforced concrete (SRC) test model column. A variety of different working conditions are artificially set in the model to simulate various defects that may occur in real engineering components. The stress wave signals under different detection paths are analyzed to verify the feasibility and effectiveness of the structural health detection method based on piezoelectric technology and weight analysis and the new damage index in the full-scale structural detection project.

## 2. Internal Defect Detection Principle of Steel-Reinforced Concrete Based on Piezoelectric Technology and Weight Analysis

### 2.1. Stress Wave Analysis Based on the Principle of Wave Method

**Figure 2:**
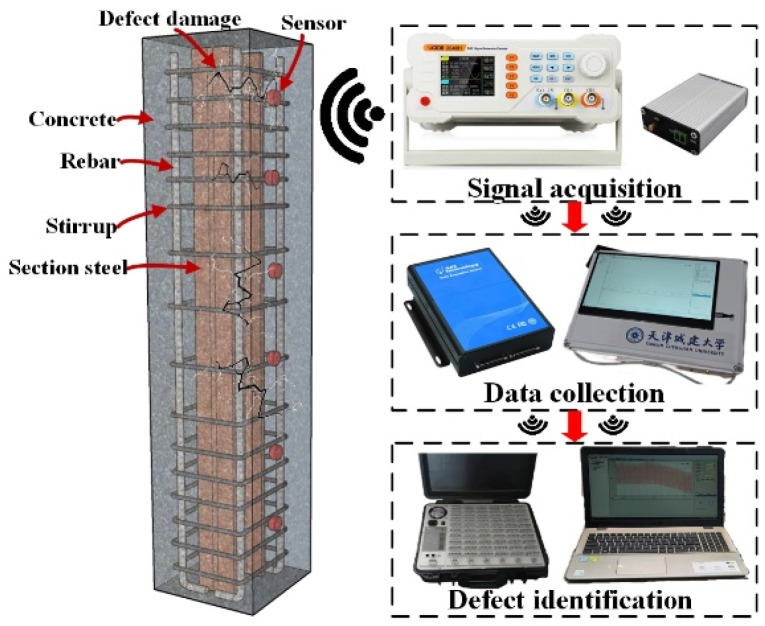
The schematic diagram of defect recognition principle of fluctuation method.

The wave propagation method uses the positive and inverse piezoelectric effects of piezoelectric ceramic materials to arrange the actuators and sensors at both ends of the structure to be tested to establish an excitation-sensing channel. The structural defect detection technology based on the piezoelectric wave method is shown in Figure 2. The basic principle is that two or more piezoelectric sensors are attached to the surface of the specimen or buried inside to form a sensing array. The stress wave generated by the actuator is received by the surrounding corresponding sensors, and the stress wave voltage signal is output. When the stress wave propagates from the concrete to the air, due to the extremely large difference in acoustic impedance (Z = ρc) between the two media (Z_concrete_ ≈ 8 × 10^6^ Pa·s/m; Z_air_ ≈ 400 Pa·s/m), the stress wave transmission coefficient approaches zero [27]. Therefore, the stress wave propagates in the concrete, and the existence of the defect will change its original propagation path so that the defect can only be bypassed by diffraction. Diffraction will increase the diffusion angle of the incident stress wave after it passes through the defect. The amplitude and frequency characteristics of the diffracted wave can indirectly reflect the size of the defect. Larger defects will lead to more significant attenuation of the diffraction signal, reduce the stress signal received by the sensor, and generate a smaller voltage response. By transmitting and receiving signals, the structural state in a large range can be detected. At the same time, by analyzing the difference in stress wave signals before and after structural damage, the characteristic parameters carrying damage information can be extracted, and the structural state information can be grasped to realize structural defect detection [28].

### 2.2. Wavelet Packet Energy Correction Based on Band Energy Ratio and Entropy Normalization

The wavelet packet transform developed on the basis of orthogonal wavelet can decompose the low-frequency part and the high-frequency part of the signal at the same time. According to the characteristics of the target signal, it is decomposed into multiple equal-bandwidth component signals, and the two high-frequency sub-bands and low-frequency sub-band nodes decomposed at the first layer are recursively decomposed to obtain four sub-nodes. After N-dimensional wavelet packet decomposition and reconstruction, the sub-signals with 2^N^ different frequency bands at the last layer are obtained [29]. After wavelet packet decomposition, the energy vector of sub-signals in each frequency band of the last layer is defined as:

(1) $$S_{K}=\left\{e_{k,1},e_{k,2}\ldots e_{k,2^{N-1}},e_{k,2^{N}}\right\}$$

(2) $$e_{k,i}=\sum_{j=1}^{M}{\left|s_{k,i,j}\right|}^{2}$$

where *S_k_* is the sub-signal of each frequency band at the end layer after wavelet packet decomposition; *e_k_*_,*i*_ is the energy value of each sub-signal generated by the original signal after multiple wavelet packet decompositions; *M* is the sampling number of the original signal; and *s_k_*_,*i*,*j*_ are the amplitude data of the bottom signal at different nodes. Therefore, the wavelet packet energy value of the original detection signal is:

(3) $$E_{total}=\sum_{k=1}^{2^{N}}e_{k,i}$$

However, the wavelet packet energy directly reflects the strength of the time-domain signal. In a scene with high-frequency noise energy, directly calculating the wavelet packet energy value of the original detection signal may cause misjudgment of the feature extraction of the damaged signal contained therein. In order to more reasonably reflect the contribution of different frequency band sub-signals and measure the degree of frequency band energy distribution, this study proposes a comprehensive correction coefficient method based on frequency band energy ratio and entropy normalization for wavelet packet energy analysis to process the original detection signal. Firstly, the sub-band energy ratio and the normalized correction coefficient based on the band energy ratio are calculated by Equations (4) and (5):

(4) $$\rho_{j,k}=\frac{\sum_{j=1}^{M}{\left|s_{k,i,j}\right|}^{2}}{\sum S_{k}}$$

(5) $${\rho^{\prime }}_{j,k}=\frac{\rho_{j,k}}{\sum_{j=1}^{M}\rho_{j,k}}$$

where *ρ_j_*_,*k*_ is the proportion of sub-band signal energy decomposed by wavelet packet; and *ρ′_j_*_,*k*_ is the normalized correction coefficient based on the frequency band energy ratio.

The energy proportion of the sub-band can highlight the high-energy quantum band containing effective features, but it cannot distinguish the energy source (including effective signals or noise). Because the energy of the background noise is dispersed and the entropy value is high, the higher the entropy, the more dispersed the energy. To avoid the influence of high-frequency noise on the effective feature extraction of signals, energy entropy is introduced to measure the degree of confusion in the energy distribution of the frequency band. Firstly, the sub-band energy distribution entropy is calculated, and then the energy entropy is inversely weighted to reduce the signal energy weight caused by high-frequency noise and suppress noise interference. Based on the entropy normalization, the correction coefficient is obtained as follows:

(6) $$H_{S}=-\sum_{k=1}^{2^{N}}\left(\frac{S_{k}}{{\sum S}_{k}}\mathrm{lg}\frac{S_{k}}{{\sum S}_{k}}\right)$$

(7) $$\varepsilon_{j,k}=e^{-\lambda H_{j,k}}(\lambda >0)$$

(8) $${\varepsilon^{\prime }}_{j,k}=\frac{\varepsilon_{j,k}}{\sum_{j=1}^{M}\varepsilon_{j,k}}$$

where *H_S_* is the sub-band signal energy entropy decomposed by wavelet packet; and *ε′_j_*_,*k*_ are normalized correction coefficients based on signal energy entropy. Combined with the hybrid method of energy ratio and entropy value, the mathematical expression of the composite correction coefficient is proposed as follows:

(9) $$\sigma_{j,k}={\rho^{\prime }}_{j,k}\cdot {\varepsilon^{\prime }}_{j,k}$$

(10) $$\hat{E}=\sigma_{j,k}\cdot E_{total}$$

where *σ_j_*_,*k*_ is the energy correction coefficient of the hybrid method combining energy ratio and entropy normalization; and *Ê* is the corrected wavelet packet energy value. The composite correction coefficient is designed by frequency band energy ratio and entropy normalization to realize multi-dimensional feature fusion of signals. In the case of high noise energy but chaotic distribution in the construction site, the composite coefficient highlights the key frequency band of stress wave impact and suppresses high-frequency noise. The weight of the noise frequency band is reduced by entropy inverse weighting to avoid misjudgment caused by relying solely on the energy ratio. At the same time, the entropy value is used to reflect the energy distribution characteristics, and the signal energy stability caused by amplitude fluctuation sensitivity is optimized while enhancing noise immunity.

### 2.3. Basic Principle of Damage Index Based on Weight Influence Coefficient

In the structural health monitoring technology based on spectral analysis, the identification of structural damage can be realized by comparing the change in vibration response spectrum under damage state and health state. In order to quantitatively evaluate the degree of structural damage, many scholars use statistical indicators to calculate the relative variation of stress wave time-domain signal characteristics. At present, the typical damage metrics proposed and commonly used include RMSD, MAPD, and CCD, which are expressed as follows:

(11) $$RMSD=\sqrt{\frac{\sum_{i=1}^{N}{\left(E_{i}-{E_{i}}^{H}\right)}^{2}}{\sum_{i=1}^{N}{\left({E_{i}}^{H}\right)}^{2}}}$$

(12) $$MAPD=\sum_{i=1}^{N}\left|\frac{E_{i}-{E_{i}}^{H}}{{E_{i}}^{H}}\right|$$

(13) $$CCD=1-\frac{\sum_{i=1}^{N}\left(S_{i}-\bar{S_{i}}\right)\left({S_{i}}^{H}-\bar{{S_{i}}^{H}}\right)}{\sqrt{\sum_{i=1}^{N}{\left(S_{i}-\bar{S_{i}}\right)}^{2}\sum_{i=1}^{N}{\left({S_{i}}^{H}-\bar{{S_{i}}^{H}}\right)}^{2}}}$$

These damage metrics are obtained by systematically comparing the differences between the ‘health reference spectrum’ and the ‘damage state spectrum’ [30,31]. Their mathematical essence is to quantify the correlation and deviation between the two spectral curves. When the structural health state changes, the changes in various response spectrum characteristics, such as resonance frequency shift, spectral peak splitting, and new resonance points in the response spectrum of the stress wave time-domain signal, will change the value of the damage metric [32]. Therefore, this study introduces the weight influence coefficient to establish a regression model of wavelet packet energy value and damage index calculated from the stress wave signal of the piezoelectric sensor [33]. The mathematical expression of the regression model is shown in Equation (14):

(14) DI=αX+βY+γZ

where *DI* is the defined total damage evaluation index; *X* is the root mean square deviation between the target detection path based on the wavelet packet energy value and the health state; *Y* is the average absolute percentage deviation between the detected target energy value and the healthy state energy value; *Z* is the cross-correlation coefficient deviation between the detected target energy value and the health state energy value; and *α*, *β* and *γ* are the undetermined weight influence coefficients. After the regression model is established, AHP (analytic hierarchy process) is introduced to decompose multiple damage metrics into independent and logically related sub-targets. Combining qualitative and quantitative analysis, combined with the empirical judgment of decision-makers, through the weighting of hierarchical structure, the subjective experience judgment is combined with mathematical calculation to finally determine the weight of each damage factor in structural detection damage identification. The comparison scale is set by the 1–9 scale method and the comparison matrix is constructed as shown in Table 1.

Calculate the eigenvector and eigenvalue of the judgment matrix row vector, and normalize the eigenvalue as follows:

(15) λi=∏i=1n αijn

(16) $$\lambda =\frac{\lambda_{i}}{\sum \lambda_{i}}$$

where *a_ij_* is the element in the weight comparison judgment matrix; *λ_i_* is the geometric mean of the row matrix elements; and *λ* is the normalized weight value of each damage factor. According to Equations (15) and (16), the corresponding weight coefficients of RMSD, MAPD, and CCD are 0.258, 0.637, and 0.105, respectively. In order to ensure the objectivity and comprehensiveness of the decision-making process and avoid decision-making errors caused by human subjectivity and one-sidedness, the maximum eigenvalue *λ*_max_ is used to calculate the random consistency index (CI) and the consistency ratio index CR, and the consistency test is performed at each level when single-criteria ranking is performed. The calibration consistency test threshold is 0.1, as shown in Equations (17)–(19):

(17) $$\left(\begin{array}{ccc} 1 & \frac{1}{3} & 3\\ 3 & 1 & 5\\ \frac{1}{3} & \frac{1}{5} & 1 \end{array}\right)\times \left(\begin{array}{c} 0.258\\ 0.637\\ 0.105 \end{array}\right)=\left(\begin{array}{c} 0.804\\ 1.966\\ 0.318 \end{array}\right)$$

(18) $$CI=\frac{\lambda_{\max}-n}{n-1}=\frac{1.966/0.637-3}{2}=0.0432$$

(19) $$CR=\frac{CI}{RI}=\frac{0.0432}{0.58}=0.0744<0.1$$

where RI is the random consistency index correction value, and the RI is referred to in Table 2. The consistency ratio index (CR) < 0.1 is calculated by Equation (19), and the calculated and normalized feature vector can be regarded as the weight vector of each damage factor; that is, *α* = 0.258, *β* = 0.637, and *γ* = 0.105.

## 3. Study on the Performance of the Defect Detection Method in Concrete Specimen Based on Piezoelectric Technology and Weight Analysis

### 3.1. Structure and Preparation of the Piezoelectric Sensor

**Figure 3:**
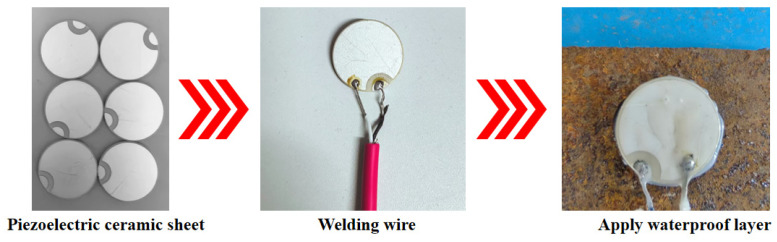
Piezoelectric sensor production process.

Thicker piezoelectric ceramics require low-frequency signals with larger voltage amplitudes for excitation. However, due to the longer wavelengths, low-frequency signals tend to bypass slightly damaged interface areas, resulting in the loss and misjudgment of effective detection information. Therefore, R25 mm × 2 mm^3^ PZT wafers with 5A-type flip electrodes are selected as the driving and sensor functions in the experiment, and the specific performance parameters are shown in Table 3. The piezoelectric sensor is made of PZT wafer, epoxy resin, and shielded wire, and the preparation process is shown in Figure 3. The positive and negative poles of one side of the piezoelectric ceramic are respectively connected to the double-core shielded wire. The wires are connected to the positive and negative poles of the piezoelectric ceramic disc by soldering with an electric soldering iron. During the soldering process, the size of the weld points should be kept uniform and the piezoelectric ceramic should be avoided from being damaged. After the welding is completed, select piezoelectric sensors with good parameters such as capacitors and resistors for use. When making the test piece, to prevent the piezoelectric ceramic from being crushed or damaged during use or water entering the weld points causing short circuits, and to ensure the stable transmission of stress wave signals, the piezoelectric ceramic sensor and the weld points and other energized parts need to be wrapped with epoxy resin for waterproof treatment before embedment [34]. Finally, use a multimeter to check the circuit condition and prevent leakage and other phenomena.

### 3.2. Experimental Design and Specimen Making

**Figure 4:**
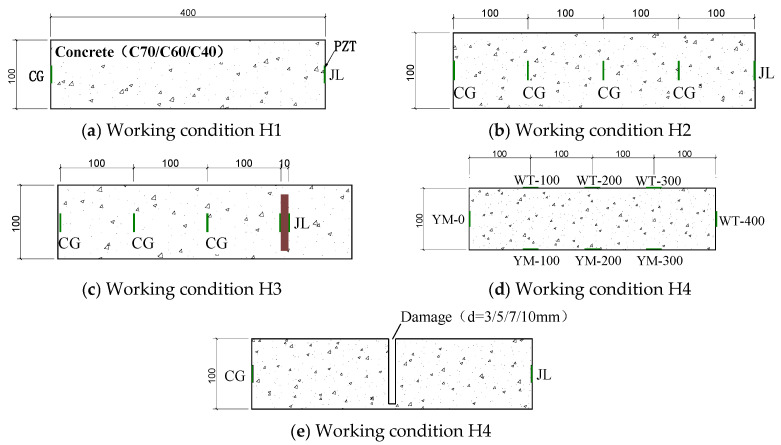
Specimen section and working condition setting.

**Figure 5:**
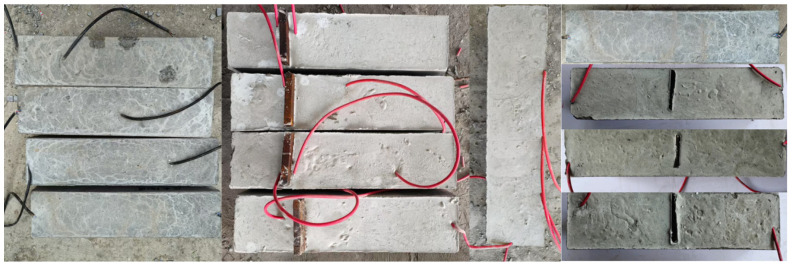
Concrete specimen.

There are many factors affecting the signal propagation of piezoelectric ceramic sensors in steel-reinforced concrete. In order to explore the influence of various working conditions on signal propagation, five groups of 100 × 100 × 400 mm concrete test blocks were made to simulate the internal detection conditions of the structure. In this study, a total of five working conditions were designed. The working condition H1 is a non-defective test block with different concrete strength grades, and the mechanical properties of concrete are shown in Table 4. Because the steel-reinforced concrete structure is widely used in super high-rise buildings, high-performance concrete is often used as the bearing member in practical engineering. Therefore, concrete with a strength grade of C70 is used to carry out the residual condition test. H2 is a defect-free test block with different detection distances. Working condition H3 refers to defect-free test blocks at different detection distances in the presence of steel plate obstruction. Working condition H4 is a defect-free test block with different arrangements of measuring points, and ‘YM-0’ is the signal excitation end. Working condition D5 is a defect test block with different gap widths. The details and parameters of each working condition are shown in Table 5 and Figure 4 (where ‘JL’ is the excitation end, ‘CG’ is the sensing end, ‘WT’ is the external sensor, ‘YM’ is the embedded sensor, and ‘WT/YM-100’ is the external/embedded arrangement + sensor detection distance). Except for the external piezoelectric sensor in the working condition H4, the other sensors are embedded in the concrete test block in advance for waterproof treatment. According to the design idea, the piezoelectric ceramics with good waterproof function are arranged inside the concrete test block and pasted with epoxy resin. After the piezoelectric ceramic is arranged with epoxy resin hardening and the position is fixed to form a set of piezoelectric ceramic health detection systems, the concrete test block is poured. The single defect detection of concrete test blocks was carried out after the pouring specimens were cured under standard conditions. The test site is shown in Figure 5.

### 3.3. Test Monitoring System

**Figure 6:**
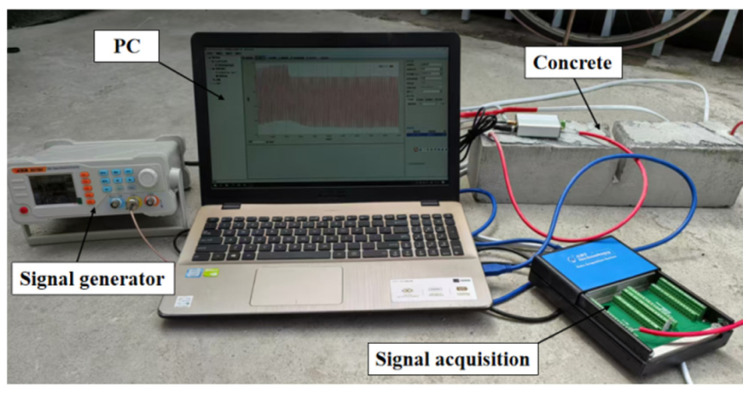
Piezoelectric ceramic active damage detection system.

The damage detection system is shown in Figure 6. The piezoelectric ceramic sensor is excited by a sinusoidal linear sweep signal. The frequency is set to 1~30 KHz and the voltage amplitude is 10 V by pre-sweeping. In order to improve the signal-to-noise ratio and increase the intensity of stress wave propagation, the excitation voltage signal is amplified, and the voltage amplification factor is set to five times. The sampling frequency is set to 1 M/s, and the continuous sampling mode is used to obtain complete and accurate electrical signal data [35]. Based on the wavelet packet energy theory, the stress wave output signal of the piezoelectric sensor under various working conditions is decomposed into wavelet packet energy of multiple nodes, with db3 as the wavelet basis. The wavelet packet energy value of the corresponding detection path is calculated by reconstruction, and the signal strength received by the sensor is characterized by the wavelet packet energy value. All the detection paths were sampled three times, and the average value of the three stress wave signal detection results was taken for data analysis.

**Figure 7:**
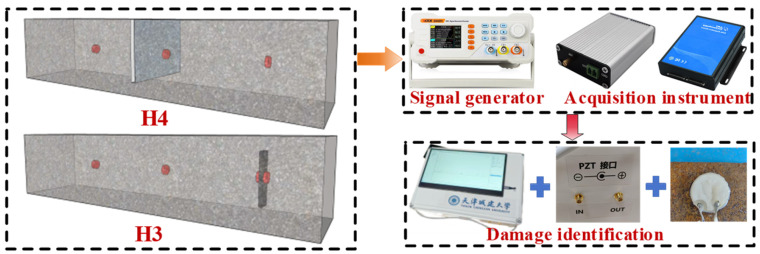
Composition of the defect damage identification instrument.

The process of structural quality detection includes three aspects: signal excitation, signal acquisition, and damage identification. At present, the use of piezoelectric ceramics to detect the internal damage process of concrete has problems, such as instrument dispersion, complex wiring, and poor synchronization in the detection system [36]. Aiming at the problems of scattered signal excitation and acquisition equipment, low efficiency of multi-instrument cooperative operation, and signal attenuation and interference caused by complex on-site wiring in traditional building health monitoring systems, this study proposes an integrated monitoring equipment optimization scheme based on hardware integration and signal cooperative control. Through the modular integrated design of the signal generator, power amplifier, and data acquisition instrument, an integrated device with excitation-amplification-acquisition full link function is constructed, which significantly reduced the volume of the equipment and greatly improved the deployment efficiency and system reliability in the field environment, as shown in Figure 7. At the hardware level, the FPGA + ARM dual-core architecture is adopted, with 5 basic waveforms and 32 arbitrary waveforms built in to meet the output requirements of the excitation signal. The power amplifier module introduces adjustable gain adjustment technology, which can achieve the maximum output voltage peak-to-peak value of 130 Vpp in the frequency range of 0–40 kHz. The data acquisition module integrates a 16-bit high-precision ADC and anti-aliasing low-pass filter. The sampling rate can reach up to 1 M/s.

## 4. Test Result Analysis

### 4.1. Concrete Grades

**Figure 8:**
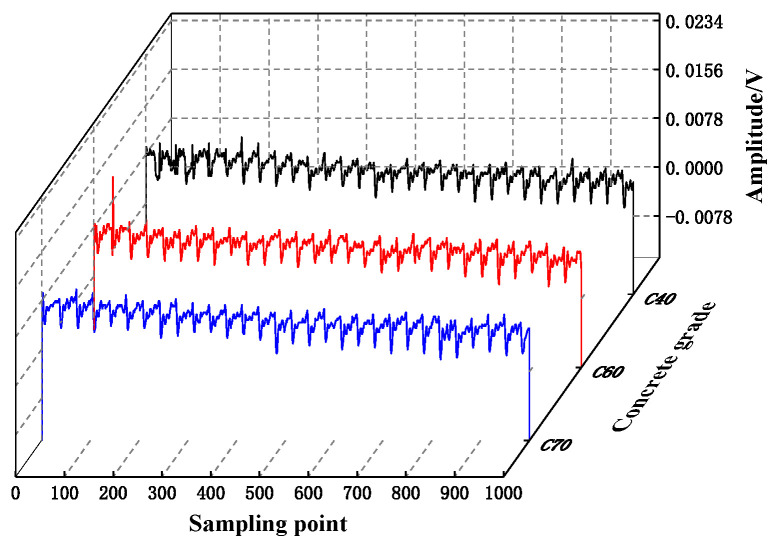
The time-domain signal of H1 stress wave.

In the construction stage of the project, concrete of different strength grades will be selected for different stress components. In order to verify the universality of the detection method of the internal defects of concrete structures based on piezoelectric technology, the propagation law of stress wave in concrete of different strength grades is explored, and the results of stress wave signals collected under working condition H1 are analyzed, as shown in Figure 8. It can be seen from Figure 8 that the stress wave signal stacking range under different strength grades of concrete is almost overlapped when the piezoelectric ceramics are excited under the same driving conditions. The voltage values collected by the piezoelectric sensor are basically the same. The maximum amplitude of the signal voltage collected by the piezoelectric sensor in the three strength grades of C70, C60, and C40 is 0.00987 V, 0.01092 V, and 0.0106 V, respectively. The maximum amplitude of the signal voltage under the C70 condition ranges from about 9.62% to 6.89%, compared with the C60 and C40 conditions, which are less than 10%, indicating that the concrete strength grade has little effect on the propagation of the piezoelectric ceramic stress wave. It is indicated that within the intensity range of C40 to C70, the response difference of the piezoelectric signal is controllable. This indicates that the structural health detection method based on piezoelectric ceramic active sensors can be applied to the detection of concrete structures within the strength range of C40 to C70 [37].

### 4.2. Detection Range

**Figure 9:**
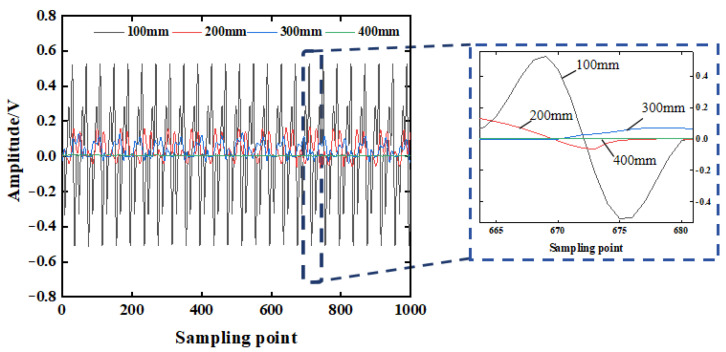
The time-domain signal of the H2 stress wave.

As the detection distance increases, due to the scattering effect of the wave in the concrete, the stress wave released from the excitation end will diffuse and attenuate in the concrete structure. The stress signal received by the sensor decreases, and a smaller voltage response will be generated. In order to explore the attenuation law of the stress signal with the change in the detection distance, the voltage response obtained by the piezoelectric sensor under different detection distances is analyzed, as shown in Figure 9. It can be seen from Figure 9 that the amplitude of the stress wave signal decreases with the increase in the detection distance. When the propagation distance is 100 mm, the effective signal of the stress wave can be detected obviously. When the distance is 400 mm, the stress wave signal with an amplitude of 0.01251 V can still be collected.

**Figure 10:**
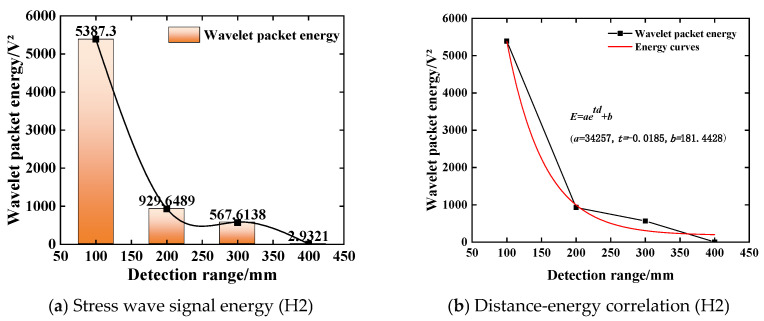
The change rule of the stress wave signal (H2).

The stress wave signal collected by the piezoelectric sensor is non-stationary. In order to better reflect the time-domain localization characteristics of the short-term high-frequency signal, the time-domain analysis of the voltage amplitude signal is carried out based on the wavelet packet energy theory. The wavelet packet energy value of each stress wave signal at different detection distances is calculated by the custom function of the MATLAB (R2022b) wavelet packet toolbox. The energy value calculation result is shown in Figure 10a. From Figure 10a, it can be seen that the energy value of the stress wave signal decreases with the increase in detection distance. When the detection distance is 400 mm, the corresponding wavelet packet energy value is 2.9321 V^2^, which is reduced by about 92.94% compared with the detection distance of 100 mm. This shows that under the condition of no defect, when the detection distance is 400 mm, the stress wave will produce a large loss in the concrete structure. The energy of the stress wave signal obtained by the piezoelectric sensor is relatively small, which is consistent with the variation law of the signal voltage amplitude. Considering the comprehensive anti-interference ability and signal acquisition sensitivity, the distance between the excitation end and the acquisition end of the piezoelectric ceramic sensor is controlled within 400 mm [23]. The signal wavelet packet energy values at different distances are fitted by curve fitting, and the approximate fitting result of the detection distance-wavelet packet energy value relationship is obtained. The curve fitting determination coefficient R^2^ is 0.9819, and the fitting result is shown in Figure 10b. The fitting curve is:

(20) $$E=3.4257\times 10^{4}e^{-0.0185d}+181.4428$$

where *d* is the distance between the excitation end of the piezoelectric sensor and the acquisition end; and *E* is the wavelet packet energy value of the stress wave signal at different detection distances.

### 4.3. Steel Plate Barrier

**Figure 11:**
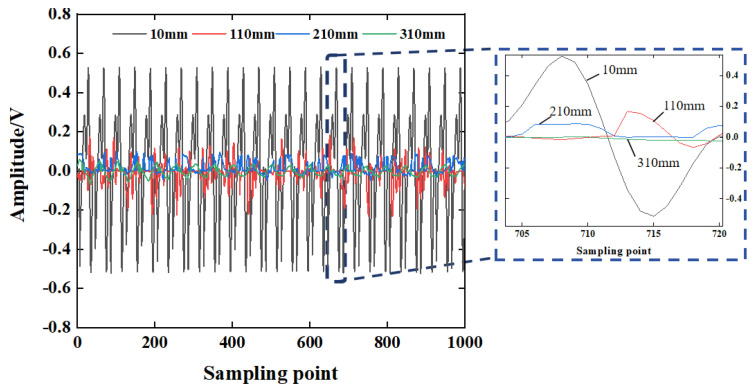
The change rule of the stress wave signal.

The steel-reinforced concrete structure is subjected to the external load jointly by the steel-reinforced column and the concrete. There is a stiffener between the signal excitation end and the sensing end to block the propagation of the stress wave in the concrete. Due to the different media in the propagation process, the stress wave waveform distortion, the propagation path, and the propagation energy change. In order to obtain the propagation law of the stress wave signal in the concrete under the barrier of the steel plate, the time-domain sensing signal of the stress wave collected by the working condition H3 is selected for analysis, as shown in Figure 11. It can be seen from Figure 11 that the amplitude of the stress wave signal decreases with the increase in detection distance in the presence of the steel plate barrier. When only the steel plate is blocked, the sensor can collect a stable stress wave signal, and the signal voltage amplitude is 0.632 V. As the detection distance increases, the signal voltage amplitude is only 0.165 V when the distance between the excitation end and the sensing end increases to 310 mm.

**Figure 12:**
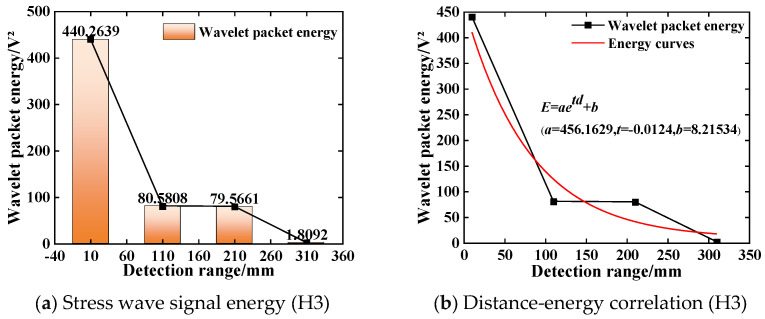
The variation law of stress wave signal (H3).

The wavelet packet energy value of each stress wave signal at different detection distances is calculated by the custom function of the MATLAB (R2022b) wavelet packet toolbox. The calculation result of the energy value is shown in Figure 12a. The stress wave signal value is 440.2639 V^2^ under only the steel plate barrier condition, and the stress wave signal energy value decreases to 1.8092 V^2^ when the detection distance increases to 310 mm. The signal wavelet packet energy values at different distances are fitted by curve fitting, and the approximate fitting result of the detection distance-wavelet packet energy relationship under the steel plate barrier is obtained. The curve fitting determination coefficient R^2^ is 0.9872, and the fitting result is shown in Figure 12b. The energy change fitting curve is:

(21) $$E=456.1629e^{-0.0124d}+8.21534$$

where *d* is the distance between the excitation end of the piezoelectric sensor and the acquisition end under the steel plate barrier condition; and *E* is the wavelet packet energy value of the stress wave signal at different detection distances.

It can be seen from Equations (20) and (21) that the signal energy decreases with the increase in the wave propagation distance, and the quantitative relationship between the stress wave energy and the propagation distance satisfies the exponential relationship. Comparing the analysis results of working condition H2, it can be seen that the stress wave signal obtained by the piezoelectric sensor under the condition of steel plate barrier is significantly reduced compared with the plain concrete condition, indicating that the transformation of steel and concrete medium during the propagation of the stress wave will lead to the reduction in voltage response obtained by the piezoelectric sensor and the attenuation of propagation energy.

### 4.4. Layout of Measuring Points

**Figure 13:**
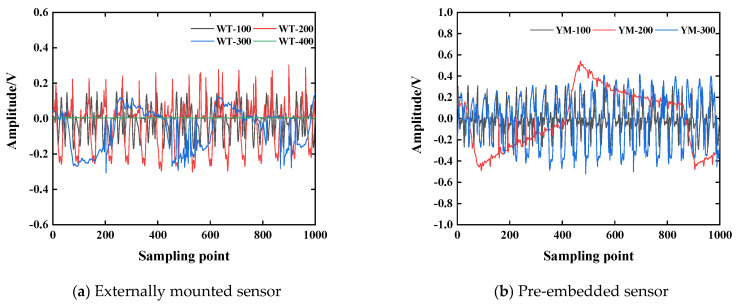
The time-domain signal of the H4 stress wave.

**Figure 14:**
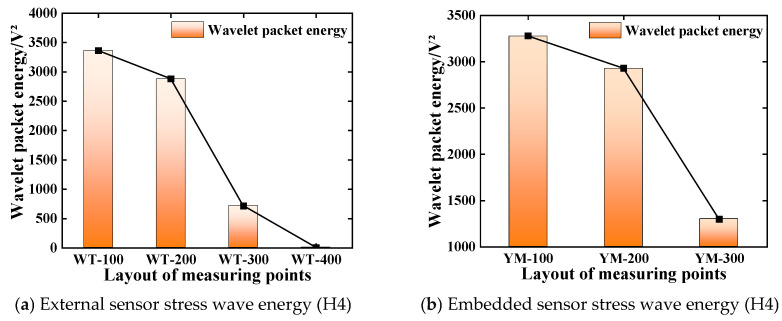
The variation law of stress wave signal (H4).

The piezoelectric sensor used in this study is made of piezoelectric ceramic wafers. The change in the incident angle of the stress wave signal will lead to a change in the voltage response of the sensor. At the same time, the external sensor mainly obtains the Lamb wave on the surface of the structure, and the embedded sensor obtains the body wave in the structure, which will also lead to a change in stress wave propagation energy. In order to explore the effect of sensor arrangement on the propagation of the stress wave signal, the test results of working condition H4 are analyzed, as shown in Figure 13. It can be seen that as the incident angle of the signal increases, the signal waveform exhibits obvious distortion and non-stationarity. In order to reflect the time-domain characteristics of the signal more intuitively, the wavelet packet energy value of each stress wave signal is calculated by the custom function of the MATLAB (R2022b) wavelet packet toolbox. The calculation results of the energy value are shown in Figure 14. It can be seen from Figure 14 that the corresponding energy values of WT-100/200/300 are 3361.6 V^2^, 2880.1 V^2^, and 714.49 V^2^, respectively, and the corresponding energy values of YM-100/200/300 are 3278.6 V^2^, 2928.9 V^2^, and 1299.1 V^2^, respectively. By comparing the detection results of the external sensor and the embedded sensor, when the propagation distance of the stress wave and the incident angle of the wave are the same, the standard deviations of the corresponding energy values of WT-100/200/300 and YM-100/200/300 are 2.47%, 1.69%, and 8.18%, respectively. The corresponding energy value of WT-400 is 3.068 V^2^, which is about a 4.45% difference from the energy value corresponding to the working condition of a 400 mm detection distance. This shows that when the stress wave propagation distance and the wave incident angle are the same, the response effect of the embedded and external sensors on the excitation signal is the same. However, compared with the signal energy corresponding to different wave incident angles, the energy of the incident angle of 45° is smaller than that of 0°. This is due to the different response mechanism of the piezoelectric sensor to receive the stress wave along the thickness direction and the length and width direction, indicating that the piezoelectric sensor made of the piezoelectric ceramic disc will have a more significant voltage response to the external load or deformation along the thickness direction than the length and width direction [38,39].

### 4.5. Defect Size

**Figure 15:**
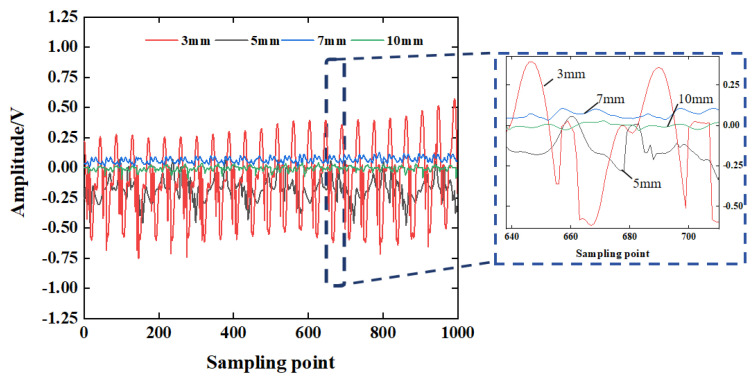
Time-domain signal of the D5 stress wave.

In order to explore the influence of the severity of concrete defects on the propagation of the stress wave, the time-domain signal of working condition D5 is analyzed, as shown in Figure 15. From Figure 15, it can be seen that compared with the voltage amplitude of the received signal of each sensor in the defect-free condition, the voltage amplitude of the received signal of each sensor in the defective condition has been greatly attenuated. As the defect size increases, the voltage amplitude of the stress wave signal decreases. The signal voltage amplitude stacking range corresponding to a gap width of 7 mm and 10 mm is relatively close. When the defect size is 10 mm, the signal voltage amplitude is 0.176 V. Compared with the signal voltage amplitude of the defect size of 3 mm, it is reduced by about 77.04%.

**Figure 16:**
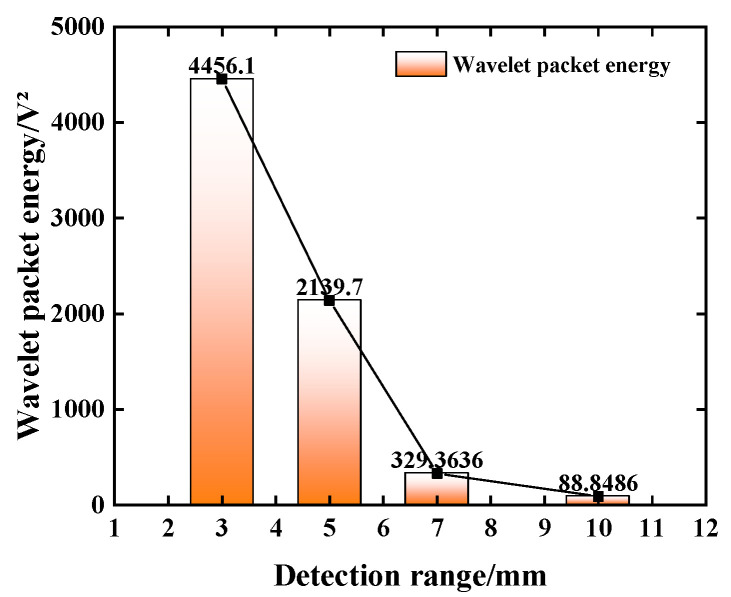
The variation law of stress wave signal (D5).

The wavelet packet energy value of the signal voltage amplitude is converted, and the calculation result is shown in Figure 16. The results show that the signal energy value decreases exponentially with the increase in the gap width. As the defect size increases, the signal energy value decays from 4456.1 V^2^ to 88.849 V^2^. At the same time, the energy values of stress wave signals with defect sizes of 7 mm and 10 mm are relatively close, which is the same as the changing trend of voltage amplitude. This shows that when the defect size is greater than 7 mm, the structure is seriously damaged, and the excitation signal cannot be projected in concrete. More stress wave signals are received by piezoelectric sensors.

**Figure 17:**
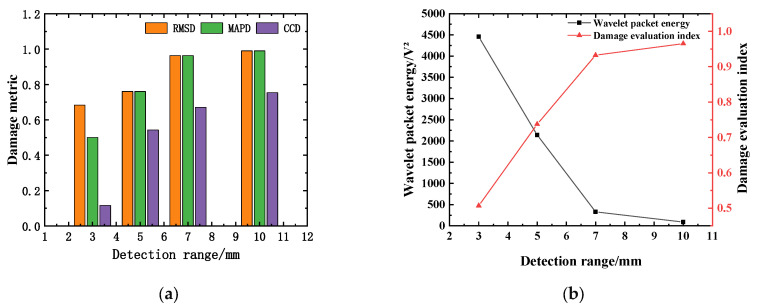
Change relationship of the damage evaluation index (DI). (**a**) Comparison of RMSD, MAPD, and CCD under different defect conditions; (**b**) the energy value and DI change with the number of obstacles.

In order to verify the effectiveness of the multi-damage factor comprehensive defect evaluation index based on the weight influence coefficient, the corresponding damage factor and defect evaluation index (DI) under different gap widths are calculated. The calculation results are shown in Table 6 and Figure 17. Figure 17a shows the comparison results of RMSD, MAPD, and CCD of the specimens under different defect conditions. It can be seen that with the increase in defect size, the three damage metrics of RMSD, MAPD, and CCD gradually increase, among which the growth rate of RMSD and MAPD is relatively consistent. The average difference between the two calculation results is 6.71%, but this is quite different from the CCD calculation results. When the defect size is 3 mm, the CCD calculation result is only 0.115, which is a 77.2% difference from the DI, indicating that only using the cross-correlation coefficient deviation as the damage index cannot identify the small damage well. Figure 17b expounds the correlation between the energy and damage index of the defective specimens and the number of obstacles. It can be seen from Figure 17b that when the defect size is 7 mm and 10 mm, the defect evaluation indexes are 0.932 and 0.965, respectively, indicating that the damage degree is extremely serious. The results are consistent with the change law of wavelet packet energy of the stress wave signal, indicating that the multi-damage factor comprehensive damage evaluation index based on the weight influence coefficient can effectively identify the internal defects of the concrete structure.

## 5. Steel-Reinforced Concrete Column Defect Detection Test

### 5.1. Specimen Design and Measuring Point Arrangement

In the test, a square concrete column with a cross-section of 0.8 × 0.8 m and a column height of 1 m was selected. Combined with the experimental study on the attenuation law of stress wave signal propagation and the construction of actual engineering projects, the internal concrete model was selected as C70. Among them, an I-shaped steel is placed in the middle of the concrete column, the steel section is H300 × 150 × 7 × 10 mm, and the column height is 1 m. Two ribs are set along the height of the column, and the ribs are 400 mm from the bottom of the steel. There is a layer of longitudinal reinforcement in the column, which is arranged in a circular direction with a diameter of 25 mm. The stirrups with a diameter of 12 mm are arranged at an interval of 0.2 m, and the overall reinforcement skeleton is formed with the longitudinal reinforcement. The rib plate divides the I-beam into two regions, and the piezoelectric ceramic plates are set up on the upper and lower sides of the rib plate. Before pouring concrete, the wooden plate is suspended at the calibration position in the mold, and the fine steel wire is used to tighten and fix it, so that the uneven medium area appears inside the concrete, to simulate the internal defects of the concrete.

**Figure 18:**
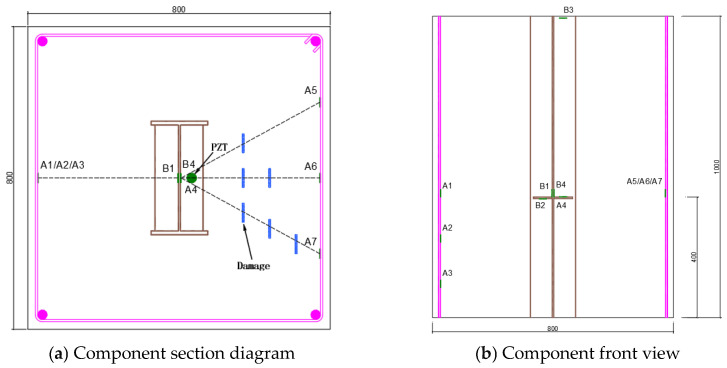
Layout of test points for steel-reinforced columns.

The arrangement of piezoelectric ceramic measuring points is shown in Figure 18a,b. Figure 18a,b shows the cross-sectional and vertical section layout of the defect damage-combined steel-concrete column, and the detected path is recorded as A-B, where A is the driving end and B is the sensing end. The detailed parameters of each working condition are shown in Table 7. No obstacles are set on the A1-B1 path, and different numbers of obstacles are set on the A5-B4, A6-B4 and A7-B4 paths respectively. The obstacle interval on the same path is 100 mm. Among them, the B2 measuring point is set at the lower part of the rib plate, the B3 measuring point is set at the upper part, and no obstacles are set between A2-B2, A3-B2, and A4-B4.

**Figure 19:**
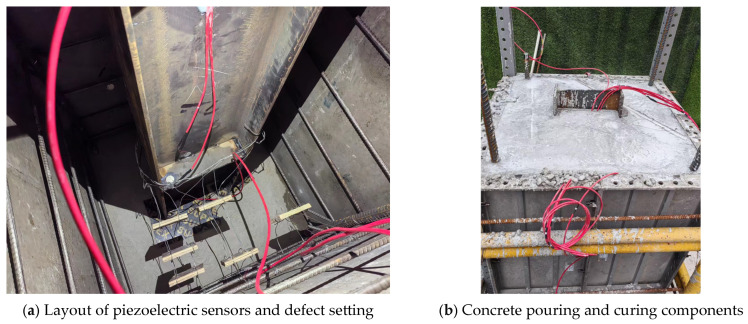
Component production process.

The piezoelectric ceramics with good waterproof function are arranged inside the steel-reinforced concrete column members according to the design idea, and the epoxy resin is still used for paste. The epoxy resin hardening is arranged in the piezoelectric ceramic, and the position is fixed to form a set of piezoelectric ceramic health detection systems. After the concrete pouring is carried out, the component model completed by pouring is placed under standard conditions. After curing, the defect detection test of the steel-reinforced concrete column is carried out. The test site is shown in Figure 19. Figure 19a is a real scene of the layout and defect settings of the piezoelectric transducer (PZT). Figure 19b shows the pouring and curing diagram of the defect damage-combined steel-concrete column. This detection test applies excitation to the piezoelectric ceramic sensor using a sinusoidal linear sweep frequency signal. The frequency is set to 1–30 KHz through pre-sweep frequency, the voltage amplitude is 10 V, and the amplification factor of the excitation voltage is 10 times. The sampling frequency is set to 1 M/s.

### 5.2. Test Monitoring Results

**Figure 20:**
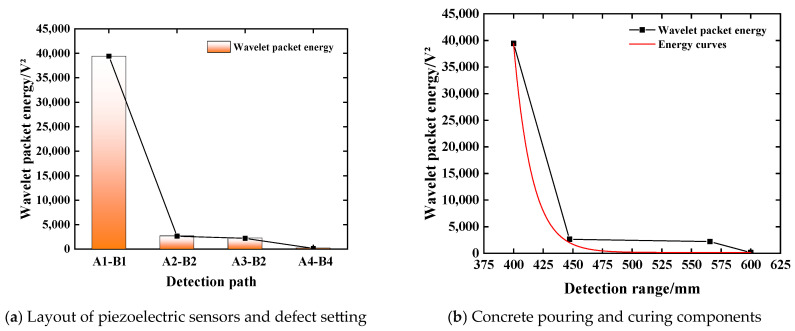
The variation law of stress wave under different incident angles and propagation distances.

Aiming at the detection of each working condition in the component model, the corresponding wavelet packet energy is calculated by the wavelet packet toolbox for the obtained stress wave signal. In order to explore the variation law of stress wave signal with the increase in incident angle and propagation distance, the voltage signals of detection paths 1–2, 3–5, 4–5, and 10–11 are taken as examples. The energy values and variation curves corresponding to the four detection paths are shown in Figure 20. The experimental results show that the stress wave energy value of the piezoelectric sensor decreases exponentially with the increase in the incident angle and the propagation distance of the wave under the combined working conditions of different incident angles and propagation distances, which is consistent with the change rule of the test data of the test block under a single working condition. It can be seen from Figure 20, that when the detection distance is 400 mm and the wave incident angle is 0° (detection path 1–2), the wavelet packet energy of the received signal of the piezoelectric sensor is 39,427 V^2^; when the detection distance is 447.21 mm, the incident angle is 27°, and when the distance is 565.69 mm, the incident angle is 45 ° (the detection paths are 3–5 and 4–5, respectively), and the wavelet packet energy value is greatly attenuated to 2641.5 V^2^ and 2204.1 V^2^, respectively. This is consistent with the results of the H4 test in the above-mentioned stress wave signal attenuation characteristics test, indicating that the piezoelectric sensor made of piezoelectric ceramic discs still exhibits a more significant voltage response to external loads or deformation along the thickness direction in the SRC model column.

**Figure 21:**
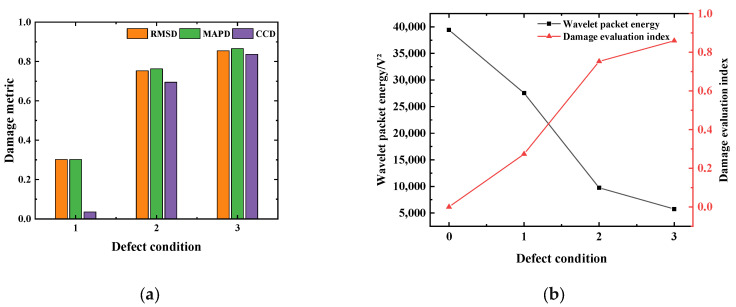
Change relationship of the damage evaluation index. (**a**) Comparison of RMSD, MAPD, and CCD under different defect conditions; (**b**) the energy value and DI change with the number of obstacles.

In order to explore the performance of the multi-damage factor comprehensive defect evaluation index based on the weight influence coefficient in the internal damage identification of steel-reinforced concrete structure, by analyzing the signal energy values of detection paths 1–2, 7–6, 8–6, and 9–6, the variation law of stress wave and the severity of quantitative damage under different defect degrees in steel-reinforced concrete model columns are revealed. By calculating the damage factor of the stress wave signal energy value under the defect condition and converting the defect damage index, the effect after conversion is shown in Table 8 and Figure 21. The damage index of the detection path under the healthy state is defined as 0. It can be found from Table 8 and Figure 21a that under one obstacle condition, the calculated value of CCD is only 0.035, which is 87.1% different from the DI value. This shows that only using the cross-correlation coefficient deviation as the defect index cannot identify the small damage well, which is consistent with the test results of the working condition D5 in the above-mentioned stress wave signal attenuation characteristics research test. This shows that the internal defect detection method of steel-reinforced concrete based on piezoelectric ceramic also shows good detection performance in structural members. This realizes the leap from the detection of plain concrete test blocks with a single working condition to the defect detection of steel-reinforced concrete structures with multiple working conditions. It can be seen from Figure 21b that the stress wave signal obtained by the piezoelectric sensor decreases with the increase in the number of obstacles on the board. The wavelet packet energy value under the three-obstacle condition is 85.42% lower than that under the barrier-free condition. The defect index increases with the increase in the number of obstacles, and the DI value gradually increases from 0 to 0.859, indicating that there are serious defects on the detection path. Therefore, the establishment of a multi-damage factor comprehensive regression model based on the weight influence coefficient can effectively characterize the damage state of the specimen and evaluate the damage level of the specimen.

## 6. Conclusions

Aiming at the complex internal working conditions of steel-reinforced concrete, this paper proposes a defect detection method based on piezoelectric technology and weight analysis. Research is conducted from aspects such as defect detection, signal processing, and damage identification. A defect evaluation index (DI) based on the weight regression model of different damage metrics is established to clarify the correlation between damage and DI. The main conclusions are as follows:

1. The multi-dimensional feature fusion of the signal is achieved by designing the composite correction coefficient through the normalization of the frequency band energy proportion and entropy value, maintaining a high noise suppression ability while retaining the effective features. The composite coefficient highlights the key frequency bands of stress wave impact and upholds high-frequency noise. The weight of the noise frequency band is reduced through entropy inverse weighting. Meanwhile, the entropy value is combined to reflect the energy distribution characteristics, enhancing the noise resistance while optimizing the energy instability caused by the fluctuation of signal amplitude;
2. The difference in stress wave signal amplitude under different concrete strength grades is less than 10%, which indicates that the structural health detection method based on piezoelectric sensors is applicable to the detection of concrete structures within the strength range of C40 to C70;
3. Based on the comprehensive test results of the test block and the SRC model column, the signal energy intensity decreases exponentially with the increase in the detection distance. The transformation of steel and concrete media during the propagation of stress waves will lead to a reduction in the voltage response obtained by the piezoelectric sensor and attenuation of the propagation energy. With the increase in the gap width and the number of obstacles, the signal energy value attenuates from 4456.1 V^2^ to 88.849 V^2^. When the defect size is greater than 7 mm, the structure is considered severely damaged, and piezoelectric sensors are unable to collect more signal energy for wave propagation;
4. Combining the influence of spectral characteristic changes on the calculated values of different damage metrics, the DI of the multi-damage factor comprehensive linear regression model based on weight analysis is proposed. The weighted defect index has more advantages in identifying minor damages compared to using CCD. The DI increases from 0 to 0.859 as the severity of the defect intensifies. The calculation results are consistent with the defect settings in the test. The effectiveness of damage evaluation indicators in the health detection of steel-reinforced concrete structures has been verified. It is also verified that when DI > 0.8, the structure is severely damaged. In practical engineering applications, this indicates significant damage inside the structure, and reinforcement measures need to be taken in a timely manner;
5. The defect detection method of steel-reinforced concrete based on piezoelectric technology and weight analysis obtained in this study still requires a large amount of on-site data verification. It is suggested that in future research, a systematic study should be conducted on the value range for defect evaluation indicators to identify the severity of internal damage in steel-reinforced concrete structures.

## Figures and Tables

**Table 1:** Damage factor weight comparison judgment matrix.

	RMSD	MAPD	CCD
RMSD	1	1/3	3
MAPD	3	1	5
CCD	1/3	1/5	1

**Table 2:** Correction value of random consistency index.

Order	1	2	3	4	5	6	7	8	9	10	11
RI	0	0	0.58	0.9	1.12	1.24	1.32	1.41	1.46	1.49	1.52

**Table 3:** Piezoelectric ceramic wafer performance parameters.

Performance Parameter	Taking Values
Electromechanical coupling coefficient	0.70
Dielectric constant ε_33_/ε_0_	2000
Dielectric loss/%	2
Piezoelectric constant d_33_/(pC·N^−1^)	450
Density/(×10^3^ kg/m^3^)	7.6
Mechanical quality factor	75
Poisson ratio	0.36

**Table 4:** The mechanical properties of the concrete.

Specification Strength	*f_ck_*/MPa	*f_c_*/MPa
C70	46.8	33.4
C60	40.1	28.6
C40	26.8	19.1

**Table 5:** Detailed parameters of each working condition.

Tag	Concrete Grades	Detection Distance/mm	Layout Form	Incident Angle of Wave	Defect Type	Defect Size/mm
H1	C70	400	Embedment	0	Zero defect	—
	C60	400	Embedment	0	Zero defect	—
	C40	400	Embedment	0	Zero defect	—
H2	C70	100	Embedment	0	Zero defect	—
	C70	200	Embedment	0	Zero defect	—
	C70	300	Embedment	0	Zero defect	—
	C70	400	Embedment	0	Zero defect	—
H3	C70	10	Embedment	0	Steel plate	—
	C70	110	Embedment	0	Steel plate	—
	C70	210	Embedment	0	Steel plate	—
	C70	310	Embedment	0	Steel plate	—
H4	C70	100	Externally bonded	45°	Zero defect	—
	C70	200	Externally bonded	27°	Zero defect	—
	C70	300	Externally bonded	18°	Zero defect	—
	C70	400	Externally bonded	0	Zero defect	—
	C70	100	Embedment	45°	Zero defect	—
	C70	200	Embedment	27°	Zero defect	—
	C70	300	Embedment	18°	Zero defect	—
D5	C70	300	Embedment	0	Gap	3
	C70	300	Embedment	0	Gap	5
	C70	300	Embedment	0	Gap	7
	C70	300	Embedment	0	Gap	10

**Table 6:** Damage metrics and defect indexes corresponding to different gap widths.

Defect Size/mm	RMSD	MAPD	CCD	DI
3	0.683	0.499	0.115	0.506
5	0.760	0.760	0.542	0.737
7	0.963	0.963	0.670	0.932
10	0.989	0.990	0.753	0.965

**Table 7:** Detailed parameters of each working condition.

Driver	Sensor	Wave Incident Angle	Number of Obstacles
A1	B1	0	0
A2	B2	27°	0
A3	B2	45°	0
A4	B3	0	0
A5	B4	0	1
A6	B4	0	2
A7	B4	0	3

**Table 8:** The corresponding damage metrics and defect indexes under different defect degrees.

Defect Condition	RMSD	MAPD	CCD	DI
0	0	0	0	0
1	0.310	0.301	0.035	0.273
2	0.752	0.762	0.694	0.752
3	0.854	0.865	0.83619	0.859

## Data Availability

Data are contained within the article.
